# Targets of Wnt/ß-Catenin Transcription in Penile Carcinoma

**DOI:** 10.1371/journal.pone.0124395

**Published:** 2015-04-22

**Authors:** Manit Arya, Christopher Thrasivoulou, Rui Henrique, Michael Millar, Ruth Hamblin, Reena Davda, Kristina Aare, John R. Masters, Calum Thomson, Asif Muneer, Hitendra R. H. Patel, Aamir Ahmed

**Affiliations:** 1 Division of Surgery, University College Hospital, London, United Kingdom and The Barts Cancer Institute, Queen Mary University of London, London, United Kingdom; 2 Research Department of Cell and Developmental Biology, The Centre for Cell and Molecular Dynamics, Rockefeller Building, University College London, London, United Kingdom; 3 Department of Pathology, Portuguese Oncology Institute and Department of Pathology and Molecular Immunology, Abel Salazar Institute of Biomedical Sciences, University of Porto, Porto, Portugal; 4 Queen’s Medical Research Institute, University of Edinburgh, Edinburgh, United Kingdom; 5 Prostate Cancer Research Centre, Division of Surgery, University College London, London, United Kingdom; 6 Dundee Imaging Facility, College of Life Sciences, University of Dundee, Dundee, United Kingdom; 7 Department of Urology, University College Hospital, London, United Kingdom; 8 Division of Surgery, Oncology, Urology and Women's Health, University Hospital of Northern Norway, Tromso, Norway; Ohio State University Medical Center, UNITED STATES

## Abstract

Penile squamous cell carcinoma (PeCa) is a rare malignancy and little is known regarding the molecular mechanisms involved in carcinogenesis of PeCa. The Wnt signaling pathway, with the transcription activator ß-catenin as a major transducer, is a key cellular pathway during development and in disease, particularly cancer. We have used PeCa tissue arrays and multi-fluorophore labelled, quantitative, immunohistochemistry to interrogate the expression of WNT4, a Wnt ligand, and three targets of Wnt-ß-catenin transcription activation, namely, MMP7, cyclinD1 (CD1) and c-MYC in 141 penile tissue cores from 101 unique samples. The expression of all Wnt signaling proteins tested was increased by 1.6 to 3 fold in PeCa samples compared to control tissue (normal or cancer adjacent) samples (p<0.01). Expression of all proteins, except CD1, showed a significant decrease in grade II compared to grade I tumors. High magnification, deconvolved confocal images were used to measure differences in co-localization between the four proteins. Significant (p<0.04-0.0001) differences were observed for various permutations of the combinations of proteins and state of the tissue (control, tumor grades I and II). Wnt signaling may play an important role in PeCa and proteins of the Wnt signaling network could be useful targets for diagnosis and prognostic stratification of disease.

## Introduction

Penile cancer (PeCa), an aggressive squamous cell carcinoma, is associated with significant morbidity and mortality in some areas of the developing world. PeCa incidence varies from 3 up to 8.3 per 100,000 men in developing nations in Asia, Africa and South America; PeCa is the most commonly diagnosed malignancy in men in Uganda [1,2]. In contrast, PeCa is rare in Europe and North America (age standardised incidence of 0.3–1 per 100000 men [1,3]). Little is known about the molecular mechanisms of carcinogenesis of PeCa; mutations in p53 and ras, and dysregulation of proteins such as cyclin D1 (CD1), E-cadherin and matrix metalloproteinase (MMP) 9 have been identified as factors involved in PeCa. Also, p53 over-expression is thought to be associated with tumor progression [4]. Mentrikoski *et al* [5], using conventional semi-quantitative scoring methods [6], have suggested that p53, CD1 and EGFR over-expression may contribute towards carcinogenesis; CD1 expression has also been co-related with tumor differentiation [7]. In a large sample cohort (125 patients) E-cadherin and MMPs 9 and 2, were found to be putative prognostic markers of PeCa [8]. Mutation in c-rasHa gene [9] and activity of cyclo-oxygenase 2 (COX2) [10] have been associated with metastatic PeCa. Despite these efforts, research related to PeCa is still in its infancy compared to other male genitourinary malignancies; this is largely due to the rarity of the disease and a paucity of research tools (human tissue or cell lines or mouse models) available to investigate PeCa. There is also a dearth of robust biomarkers of PeCa prognosis or progression.

The Wnt signaling pathway is considered a key molecular cascade for cell fate and cell proliferation during embryogenesis in animals and throughout their lifespan. Consequently, dysregulation in Wnt signaling, because of mutations or via temporal and kinetic defects in function, are causative or associated with a variety of diseases, including cancers [11–14]. Activation of Wnt signaling in cancer cell lines activates calcium release that depolarizes the nuclear membrane and facilitates the trans-nuclear translocation of ß-catenin [15]. Once translocated into the nucleus, ß-catenin activates transcription of a number of TCF/LEF responsive genes, including proto-oncogenes such as CD1 and c-MYC [16] and MMP7 [17]. Due to activation of these and other targets [16], Wnt signaling is considered a critical step in carcinogenesis in many carcinomas [18–21], including cancers of the pelvic region (e.g. colon, ovary and prostate) [11,22,23]. Targets of ß-catenin transcription are also over-expressed in many squamous cell carcinomas [24–26] (see [14] for a review). Thus, Wnt signaling proteins have been proposed as putative biomarkers for prostate and other cancers [6,23,27–29].

Little information exists on the role that Wnt signaling might play in PeCa (perhaps other than the expression of CD1, a target of ß-catenin transcription [7]) and there have been no systematic, quantitative analyses of Wnt signaling related proteins in PeCa tissue. We therefore asked the question whether elements of Wnt signaling, particularly the targets of Wnt-ß-catenin transcription, are dysregulated in PeCa?

As a first step towards a full characterization of Wnt signaling in PeCa, we chose CD1, MMP7 and c-MYC as these are transcriptional targets of ß-catenin/TCF/LEF activation and WNT4, a ligand protein and one of the members of the 19 protein WNT family. We used a multi-fluorophore labelled, quantitative immunofluorescence [6] technique, in combination with high throughput and confocal imaging and unbiased fluorophore signal quantification, to investigate the expression and co-localization of Wnt signaling targets, WNT4, CD1, MMP7 and c-MYC in penile carcinoma and normal samples. We show, for the first time, that (i) Wnt signaling proteins are up-regulated in PeCa (ii) there is a significant decrease in the expression of Wnt4, MMP7 and c-MYC in grade II compared to grade I tumors and (iii) quantitative differential co-localization shows significant differences between control, grade I and grade II tumor samples. Wnt signaling may play a key role in PeCa carcinogenesis and proteins involved in Wnt signaling could provide useful diagnostic and therapeutic targets for PeCa.

## Materials and Methods

### Tissue array

Penile tissue arrays (catalog number PE241, PE801 and T223) were purchased from US Biomax, Inc (MD). Clinical details and haematoxylin and eosin characteristics of the tissue samples used can be seen at- http://www.biomax.us/tissue-arrays/Penis/. Anonymized, archival, formalin fixed, normal penile tissue samples were also obtained by Mr Asif Muneer and Dr Rui Henrique and made into tissue arrays. The total number of unique samples (diameter 1.5mm) used in this study were 101 (18 normal and 83 malignant). A sample size calculation was made for differentiating between over-expression using intensity of fluorophores; (with an α and ß of 0.025 and expected difference of 200 mean gray value with standard deviation 100 and ratio of 1/3 between control and malignant groups to be 7 and 21); our actual sampling (18 control and 83 malignant) is therefore 40–25% greater than predicted by the sampling calculation. For quantitative analysis only control (18 patients; 37 tissue cores) and malignant squamous cell carcinoma (83 patients; 102 tissue cores) tissue samples were considered. Tissue cores of other pathology (e.g. squamous epithelium hyperplasia or verrucous squamous cell carcinoma) were excluded from the analysis. The tissue arrays yielded 82 grade I, 18 grade II malignant and 2 grade III tissue cores for analysis.

Pathological characterization was provided by US Biomax according to the standard grading consensus (Grade I or well differentiated to Grade IV or undifferentiated; description provided on the web page). Haemaotoxylin eosin stained samples used in this study, including those obtained from US Biomax, were also assessed by a pathologist, according to the criteria used by the College of American Pathologists, as stated in Velazquez EF *et al* [30]. Histologic grade and perineural invasion were considered more important than tumor thickness as predictor of nodal metastasis in penile squamous cell carcinoma invading 5 to 10 mm.

### Ethics approval

The US Biomax, Inc. website (http://www.biomax.us/faq.php#q10)states that: “all tissue is collected under the highest ethical standards with the donor being informed completely and with their consent”. For use of human tissue for research in our laboratory, ethical approval was given by the Joint University College London/University College London Hospital committees on the ethics of human research. Anonymized, archival, normal penile tissue samples were also obtained from Mr Asif Muneer (written informed consent for use of samples in research was obtained from the donors) and Dr Rui Henrique (institutional review board, Comissão de Ética para a Saúde–Instituto Portugues de Oncologia, Porto, waived the need for consent) and made into tissue arrays. The review board approved the use of human tissue for research, in compliance with the International Committee on Harmonization of Good Clinical Practice (ICH GCP); similar approval exists at the Portuguese Oncology Institute, Porto, Portugal.

### Immunofluorescence staining using 4 antibodies in penile tissue arrays

All staining was performed using Bond automated system using a protocol adapted from Toth and Mezey [31]; details of the staining procedure for IHC are given elsewhere [6,23]. Briefly, following heat induced epitope retrieval (HIER) with Novocastra pH6 retrieval buffer (using a de-cloaking chamber and blocking with 3% H_2_O_2_ in Tris Buffered Saline containing 0.01% Tween, TBST), tissue arrays were incubated with four antibodies, WNT4 (ab15699, Abcam), MMP7 (ab 4044, Abcam), CD1 (sc-718, Santa Cruz) and c-MYC (C3956, Sigma), sequentially, and visualised with fluorescent Tyramides-, FITC, Cy3 and Cy5 and streptavidin Alexa Fluor-405, respectively. CD1 was used at 1:200 dilution was incubated for 2 hours at room temperature (RT) and following TBST washes, goat anti rabbit peroxidase Fab (1:500 dilution) was incubated for 30 min at RT; subsequent to two further TBST washes, sections were incubated with Tyramide Cy3 for 10 min, followed by two further TBST washes and a 10 min HIER using Bond ER1 solution on Bond Automated System. MMP7 (diluted 1:100) was then visualised as above except Tyramide-FITC was employed, c-MYC (diluted 1:500) was next visualised as above except Tyramide-Cy5 was employed with Goat anti Mouse Peroxidase fab secondary. Finally Wnt4 (diluted 1:20) was stained as above except Mouse anti Goat Biotinylated (diluted 1:500) and Streptavidin Alexa Fluor-405 (diluted 1:200) was employed before washing in TBST and a glass coverslip was placed with Permafluor. Two tissue array slides (PE241 and PE801) were stained in two separate experiments; all tissue array slides used for signal and co-localization quantitation were stained at the same time and under identical conditions.

Imaging of each tissue core was performed using Zeiss Axioscan Z.1 slide scanner (Carl Zeiss) at 20x magnification. All four fluorescent signals were optimized at the start of study so as to not oversaturate the signal for each antibody. For a standardized comparison, the power of Calibri.2 LED lights and integration times of the Hamamatsu ORCA Flash4 camera (Hamamatsu Photonics) were kept constant for all samples. The imaging was performed on both slides (PE241 and PE801 plus the other TMA samples) on the same day with identical image acquisition settings. Montages of the individual TMA cores were automatically constructed from multiple 2048 x 2048 pixel 16 bit gray level images and stored locally on the acquisition computer. Composite images of whole tissue array used in this study are given in S1 Fig. For the measurement of Pearson co-localization coefficients for two proteins, some tissue cores were imaged using an Olympus FV1000 confocal system for fluorophore co-localization measurements.

### Quantitation of WNT4, MMP7, CD1 and c-MYC signal

An unbiased, automated analysis was employed to quantify the intensity of each fluorophore. Image analysis was performed automatically using an adapted ImageJ plugin, similar to one described elsewhere [6,23]. Briefly, the Plugin was designed to semi-automatically discriminate the whole TMA, largely, based on a threshold segmentation routine. Initially the lower and upper threshold parameters were set to include the median to maximum gray value of a range of samples across the whole sample set. These values were then used to perform a batch analysis of the entire sample set, where the mean gray values per TMA core were calculated.

### Quantitative co-localization of WNT4, MMP7, CD1 and c-MYC

33 tissue cores (8 control and 25 malignant cores including 11 grade I and 14 grade II samples with 4–16 ROIs for each core) were identified for areas of cancer visually on the serial H&E section of the tissue array. These areas were approximated on the fluorescently labelled tissue array and tissue cores were imaged using an Olympus FV1000 confocal system using a dry 40x 0.95 NA objective (plus a zoom of 4–6 times). Up to three different areas from each core were imaged and the resulting Olympus ‘oif’ files were imported into Huygens Professional software for image deconvolution. Deconvolved images were saved as Huygens specific (HDF5) files for each channel (excitation/emission (nm) Alexa Fluor-405 405 / 415–480, FITC = 488 / 500–550, Cy3 = 559 / 575–620, Cy5 = 635 / 645–740). The HDF5 files were imported into Huygens co-localization module and were background subtracted using ‘Gaussian minimum’ method; up to 4–16 regions of interest / image were analysed to calculate Pearson coefficient for co-localization.

## Results

### Quantitative analysis of Wnt related proteins expression in PeCa

**Fig 1:**
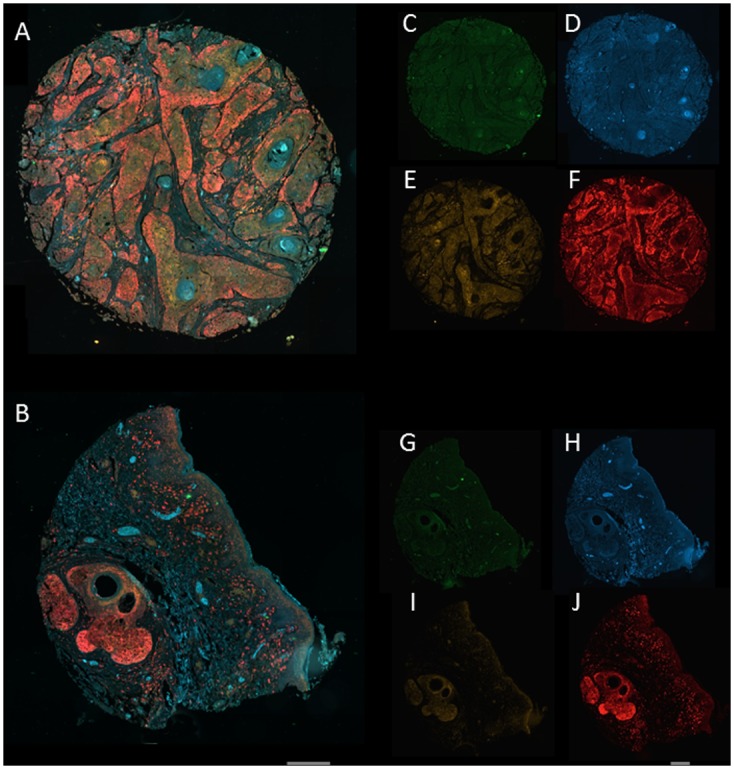
Representative images from Zeiss AxioScan Z1 slide scanner. Composite, overlay, of four fluorophores (FITC, Alexa Fluor-405, Cy3 and Cy5 for MMP7, Wnt4, CD1 and c-MYC, respectively) in (A) PeCa tissue cores (core B2 of PE241) and (B) control (core D5 of PE241, see methods). C-F are individual images used to construct the composite of PeCa tissue core, for MMP7 (C, green), Wnt4 (D, blue), CD1 (E, orange) and c-MYC (F, red). G-J are individual images used to construct the composite of control tissue core, for MMP7 (G, green), Wnt4 (H, blue), CD1 (I, orange) and c-MYC (J, red). Quantitative analysis was performed on the non-enhanced, original, gray images for which all settings were identical in all tissue core images analyzed. Scale bar = 250 μm.

All proteins (Wnt4, MMP7, CD1 and c-MYC) tested were expressed in penile tissue (Fig 1 and also S1–S2 Figs; 141 penile tissue cores from 101 unique tissue samples of which 18 were normal/cancer adjacent and 83 were malignant samples). We quantified the expression of fluorophore labelled protein (Table 1) in an unbiased manner using techniques described elsewhere [6,23];). Calculations of intensity and integrated gray value per unit area of squamous cells for each core of the four individual fluorophore labels show that the protein expression (mean) of WNT4, MMP7, CD1 and c-MYC was increased by 1.6- to 3-fold (p< 0.0001) in malignant compared to normal penile tissue (Fig 1 and Table 1).

**Table 1:** Quantitative analysis of protein expression in PeCa.

	Mean gray value	Fold increase	Significance (*p<*)
***Control CyclinD1***	2081 ± 1121		
***Malignant CyclinD1***	5037 ± 2707	2.4	0.0001
***Control MMP7***	2114 ± 902		
***Malignant MMP7***	6202 ± 2956	2.9	0.0001
***Control c-MYC***	3109 ± 1969		
***Malignant c-MYC***	4951 ± 2304	1.6	0.0001
***Control Wnt4***	3620 ± 1466		
***Malignant Wnt4***	7192 ± 2098	1.9	0.0001

The expression of fluorophore labelled proteins (CD1, Cy3; MMP7, FITC; c-MYC, Cy5; Wnt4 Alexa Fluor-405, see Fig 1) was quantified in an unbiased manner, by using a reproducible, semi-automated particle analysis protocol (see methods) using grayscale images of the stained tissue from 141 tissue cores (102 malignant and 37 non-malignant control, normal or cancer adjacent penile tissue cores). Data is presented as means ± SD for control vs malignant samples (all grades). Fold increase is relative to control for each protein and significance of difference was calculated using Mann Whitney U test.

**Fig 2:**
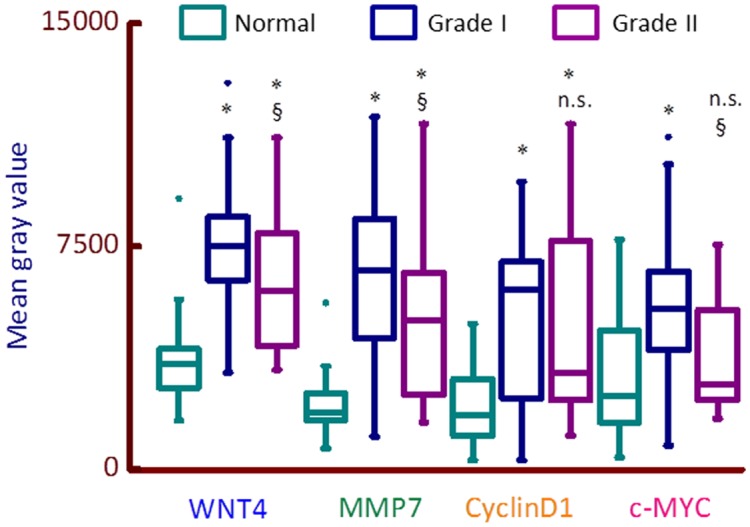
Box plot of the quantitation of expression of WNT4, MMP7, CD1 and c-MYC in PeCa tissue classified according to pathological grade. 97% of the samples used in this study were classified as grade I or II. The data presented in Table 1 was segregated into expression according to pathological grades and plotted as box plots. Filled circles are 99% value and horizontal line within the box is the median value. Protein expression (mean gray value per core) in control samples (green, n = 37); malignant tumor sample classified as grade I by a pathologist (blue, n = 82) or grade II (purple, n = 18). All proteins showed a significant difference in expression between control and malignant tumor samples. Significance of difference was measured using Mann-Whitney U test: * = p<0.001 and ^§^ = p<0.01 between control and malignant grade I or grade II samples or within grade I and grade II. There was no significant difference in protein expression for any marker between grade I and grade II samples.

The greatest increase was in the expression of MMP7 (3-fold increase in mean gray value, p<0.0001, Table 1), a key target of Wnt-ß-catenin transcription. We further classified the data based upon pathological grading of the sample (Fig 2); this analysis confirmed the significant increase in expression of all proteins in malignant tissue samples, compared to normal (control vs grades I or II). Interestingly, c-MYC expression was not significantly different between normal and grade II tumor tissue samples, although there was a significant (P<0.001) increase in grade I vs normal controls). There was also a significant decrease in expression for Wnt4, MMP7 and c-MYC in samples classified as grade II compared to grade I (Fig 2). These results identify, for the first time, that WNT4, MMP7, CD1 and c-MYC proteins are over-expressed in PeCa. These results suggest that Wnt signaling is likely to be up-regulated in PeCa and Wnt signalling proteins may be used as a biomarkers of PeCa.

### Quantitative co-localization

High magnification immunofluorescence has been employed, previously [6], to investigate changes in co-localization of proteins as a putative indicator of disease and cancer stratification. We used a similar protocol to assess if co-localization of a combination of two proteins, overexpressed in PeCa, could be used to investigate the utility of these proteins as putative biomarkers for diagnosis or for prognostic stratification of the disease.

**Fig 3:**
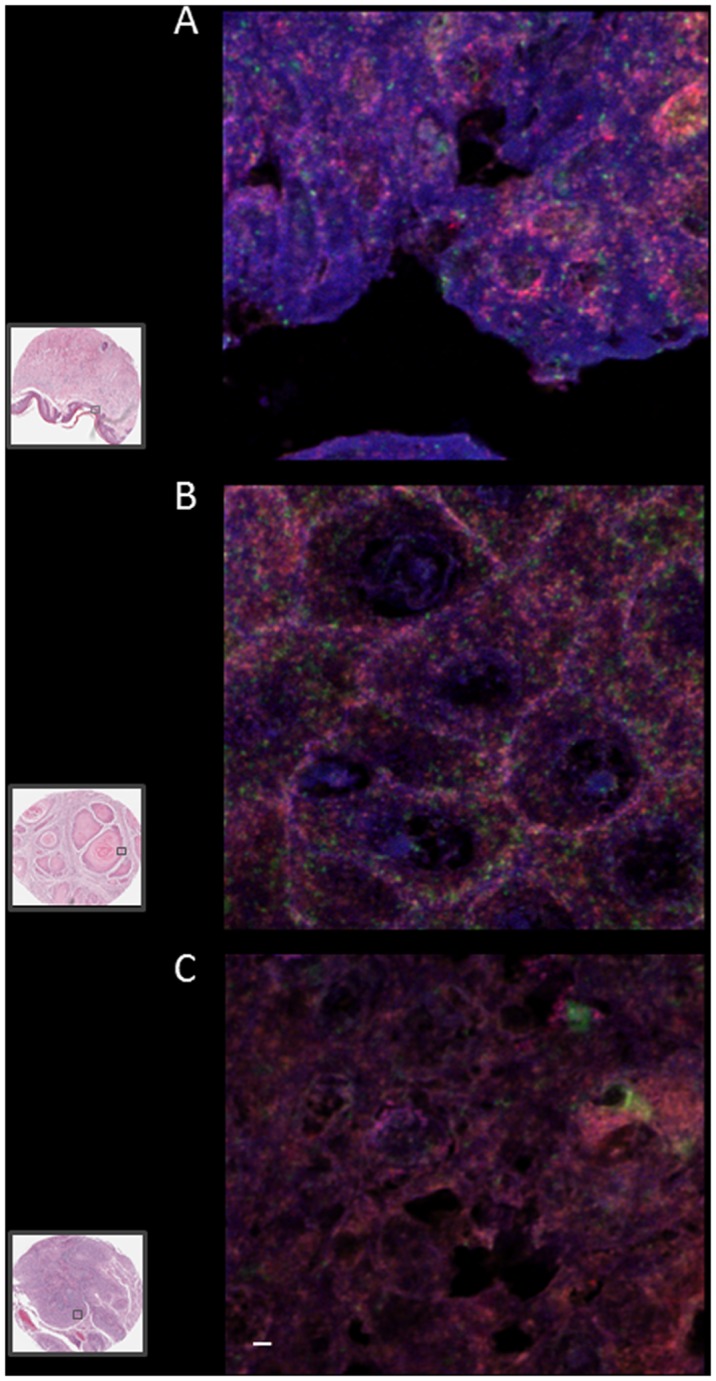
High magnification deconvolved images for WNT4, MMP7, CD1 and c-MYC co-expression in control and malignant penile tissue. Composite, representative, micrographs of immunofluorescently stained penile tissue cores used to quantify co-localization of four proteins WNT4 (Alexa 405-blue), MMP7 (FITC-green), CD1 (Cy3-orange) and c-MYC (Cy5-red) after deconvolution. Tissue cores from 8 patients (control), 25 malignant tissue cores (11 grade I and 14 grade II) were imaged using an Olympus FV1000 confocal microscope. High magnification imaging (at 1024x1024 resolution) was performed for deconvolution analysis using Huygens Profesional software. Areas of squamous cell carcinoma in malignant tissue cores were identified, visually (see box) from a serial H&E section of the tissue, and imaged at high magnification (40x with 4–6x zoom). Two representative immunofluorescently labelled cores are shown: A is control and B and C are grade I and grade II, squamous cell carcinoma samples, respectively; Inset is H&E serial sections of at low magnification. High magnification images were deconvolved to reduce noise and converted to 16bit tif files for each fluorophore (Alexa Fluor-405, FITC, Cy3 and Cy5) were imported into Huygens deconvolution software and pseudo colored (FITC, green; Alexa Fluor-405, blue; Cy3, orange; and Cy5, red). Z-projections for up to 53 images for each tissue core. Representative images of controls and malignant carcinoma samples are shown. Scale bar = 3μm. High magnification images were used for quantitative co-localization measurements.

**Fig 4:**
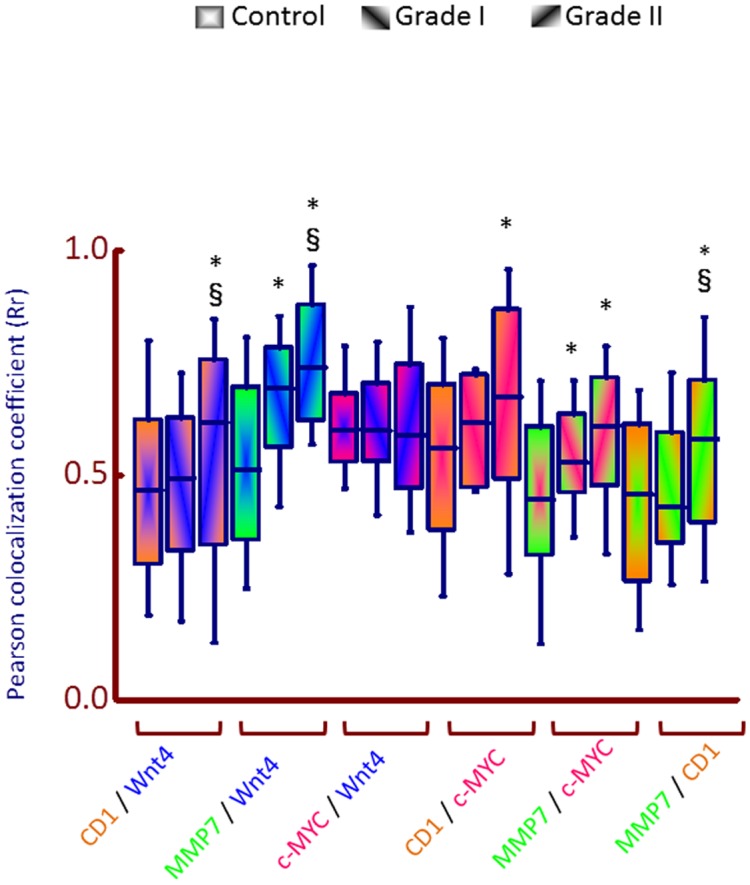
Box plot of differential pattern for WNT4, MMP7, CD1 and c-MYC co-expression in control and malignant penile tissue. Quantitative co-localization was performed from high magnification images (e.g. as represented in Fig 3) and image files used for quantitation as described in materials and methods in WCIF ImageJ software co-localization plugin to measure the Pearson coefficient of co-localization. 35 individual tissue cores that included control (gradient from centre 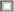) and malignant grade I (gradient from left to right 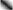) and grade II (gradient from right to left 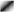) were used for each fluorophore to calculate the co-localization of expression of WNT4, MMP7, CD1 and c-MYC, generating 12 comparisons: CD1 and WNT4 (orange/blue), MMP7 and WNT4 (green/blue), c-MYC and WNT4 (pink/blue), CD1 and c-MYC (orange/pink), MMP7 and c-MYC (green/pink), MMP7 and CD1 (green/orange). Significance of difference in the Pearson coefficient of co-localization between control and grades I and II (*0.02–0.04) and between grade I and grade II (§ p<0.04, range 0.04–0.0001) samples was calculated using the Mann-Whitney U test; boxes showing comparisons without symbols are not significantly different.

Confocal images at high magnification and resolution were obtained from control and malignant samples (representative images are shown in Fig 3). The areas in malignant cores were identified visually by a pathologist, from the H&E serial section (gray box in Fig 3), prior to imaging as representative of squamous cell carcinoma. Images were deconvolved (see materials and methods) to remove noise and the resulting files were for the measurements of Pearson co-localization coefficients. This analysis (Fig 4) yielded 16 comparisons (e.g. control vs malignant grade I or grade II and between grade I and grade II samples and Wnt4 vs MMP7 and so on). Significant differences were found between the co-localization of all proteins tested in control vs malignant (either grade I or II or both) samples (Fig 4). For example, MMP7/Wnt4 showed significant differences between all comparisons (control vs grade I, control vs grade II and grade I vs grade II) whereas the Pearson co-localization coefficients were not significantly different for any comparison for c-MYC/Wnt4 combination whereas CD1/Wnt4 co-localization showed significant difference in control vs grade II but not in control vs grade I. These results indicate that, in addition to increased expression (Figs 1 and 2), alterations in co-localization of CD1 could also be a disease biomarker for PeCa and perhaps as a molecular discriminator for the grade of cancer.

## Discussion

This study makes the first, systematic, attempt to identify the role of Wnt signaling, a key pathway in carcinogenesis in many tissues, in PeCa and to identify putative biomarkers related to Wnt signaling associated with the disease.

There was a general increase in the expression of CD1, MMP7, c-MYC and WNT4, when malignant samples were compared to control (mean increase of 1.6 to 3 fold in malignant vs control, Table 1). It should be noted that although our sample size is adequate to calculate the statistical significance of these differences (see methods), the increase in the expression of the four proteins tested might be an underestimate, for example due to the maximum value for control samples for some proteins (Fig 2).

Our quantitative approach [6] also revealed that Wnt4, MMP7 and c-MYC protein expression is decreased, significantly, in grade II compared to grade I tissue (Fig 2). These results indicate that up-regulation of Wnt signalling may be critical in PeCa tumorigenesis and subsequent down-regulation of Wnt signalling may be a trigger for tumor growth and spread.

Wnt ligands, such as WNT4, activate Wnt signaling by binding to Wnt receptors and co-receptors [32] and in mammalian cells activates intracellular calcium stores that facilitates ß-catenin translocation to the nucleus [15]. WNT4 mRNA expression is elevated in fibroadenomas [33]; Li *et al* have also shown that WNT4 protein is over-expressed in pituitary adenomas [34]. Our results provide first evidence that WNT4 mediated Wnt signaling may be involved in penile squamous cell carcinoma.

MMP7 is a member of the large matrix metalloproteinase family of zinc-dependent enzymes capable of extracellular matrix degradation. It is therefore implicated in cancer metastases, particularly in squamous cell carcinoma [35,36]. Campos and colleagues [8], using the conventional, subjective expression ‘scoring’ technique, have suggested that two members of the MMP family (MMP2 and MMP9) are over-expressed in penile carcinoma. Similar to MMP7 [17], both MMP2 and MMP9 are likely targets of Wnt-ß-catenin transcription activation [37,38]. That membrane metalloproteinase targets of Wnt-ß-catenin transcription, other than MMP7, have been found to be up-regulated in PeCa support our hypothesis that Wnt signaling is likely to be active in penile squamous cell carcinoma.

G1/S specific CD1, encoded by CCND1 gene, is a modulator of cyclin dependent kinases that are involved in cell cycle co-ordination [39]. CCND1 is a human oncogene involved in a number of cancers including squamous cell carcinomas [40]. Wnt signaling is known to induce CD1 that triggers G1-phase progression and tumour cell proliferation [41]. As one of the few proteins to have been investigated in penile squamous cell carcinoma, CD1 was shown to have increased expression [7]. Our results further demonstrate that CD1 protein expression is up-regulated in PeCa, but also that co-localization of CD1 with other proteins involved in Wnt signaling could be used as putative disease biomarkers (Figs 3 and 4).

c-MYC is a proto-oncogene and a direct target of ß-catenin activated transcription [42]. It was proposed that c-MYC copy number is associated with poor outcomes in penile squamous cell carcinoma [43]. In addition to being a potent transcription factor, c-MYC, also induces expression of CD1 [44] that in turn is a target of ß-catenin induced transcription [41]. These previous observations, in conjunction with our novel observation of up-regulation of ß-catenin targets, including c-MYC in PeCa (Table 1 and Fig 2), indicate that Wnt-ß-catenin signaling may be a key component of carcinogenesis of PeCa. This is further strengthened by the observation that there is a significant difference, not only in the expression (Table 1) but also in the co-localization of c-MYC and CD1 in penile carcinoma samples compared to controls (Figs 3 and 4). It should be pointed out that gene transcription of CD1 and c-MYC is not under the control of ß-catenin/TCF/LEF activation, exclusively, but also involves other signaling pathways such as Notch and Hedgehog [45,46] and these pathways may also be activated in PeCa.

We also used co-localization as a surrogate analysis for the stratification of disease. This is based upon the rationale that a change is homeostasis may result in numerous alterations including gene and protein expression but also alterations in protein localization in the cell membranes or in the cytosol. Alterations in the topography of proteins within a cell is a feature of disease [47]. A majority of protein-protein interactions may form larger structural or functional assemblies without ever making direct contact with each other [48], thus, an assessment of co-localization of two proteins does not necessary require evidence of alterations in intimate physical interactions (e.g. via protein-protein interaction motifs). Even without any evident or predictable structural or functional significance, topographical alterations between two unrelated proteins may be utilized as a biomarker for differentiation between two conditions (e.g. normal or disease or grade I vs grade II tumor) and may prove informative for diagnostic or prognostic purposes. Our results, comparing pathological grading, indicate that such comparisons (Fig 4) could be made and that these may yield significant differences in penile carcinoma samples.

For a cancer such as PeCa for which research materials (e.g. cell lines, tissue) are in limited supply, tissue arrays from retrospective samples provide a useful tool for investigation of known cellular pathways, such as the Wnt signaling pathway. We have chosen multifluorophore labelled quantitative IHC, that we have used previously for other cancer biomarkers [6], to maximize the amount of information that could be extracted from these retrospective samples; consequently, as well as a maximizing the available resources, IHC also has limitations. For example, the status of gene structure and transcription which is difficult, although not impossible, to measure from formalin fixed samples. Also, our study has sufficient statistical power to substantiate the differences presented in this study, however, the constraints of such an approach are that other putative risk factors (e.g. HPV, smoking and others) are not considered. We hope that this study will also provide a start to conduct these studies in the future.

It has been suggested that therapies that can modulate Wnt signaling could be useful in the treatment of Wnt related diseases including a number of cancers [11,49]. The notion that Wnt signaling may be involved in penile squamous cell carcinoma is a novel observation and could provide new avenues for treating penile squamous cell carcinoma. The approach and results presented here indicate that the Wnt signalling pathway is up-regulated in PeCa and may provide a future target for diagnostic, prognostic or therapeutic intervention.

## Supporting Information

S1 Fig Example of penile tissue arrays used in this study. Tissue arrays were stained with four different antibodies and imaged using Zeiss AxioScan Z1 slide scanner. Composite, overlay, of four fluorophores (FITC, Alexa Fluor-405, Cy3 and Cy5 for MMP7, Wnt4, CD1 and c-MYC proteins, respectively) are shown.
(TIF)

S2 Fig Example of penile tissue arrays used in this study. Tissue arrays were stained with four different antibodies and imaged using Zeiss AxioScan Z1 slide scanner. Composite, overlay, of four fluorophores (FITC, Alexa Fluor-405, Cy3 and Cy5 for MMP7, Wnt4, CD1 and c-MYC proteins, respectively) are shown.
(TIF)
