# Supplementary figures and images for: Targets of Wnt/ß-Catenin Transcription in Penile Carcinoma
Source: PLoS One. 2015 Apr 22;10(4):e0124395. doi: 10.1371/journal.pone.0124395 (PMC4406530; doi:10.1371/journal.pone.0124395)

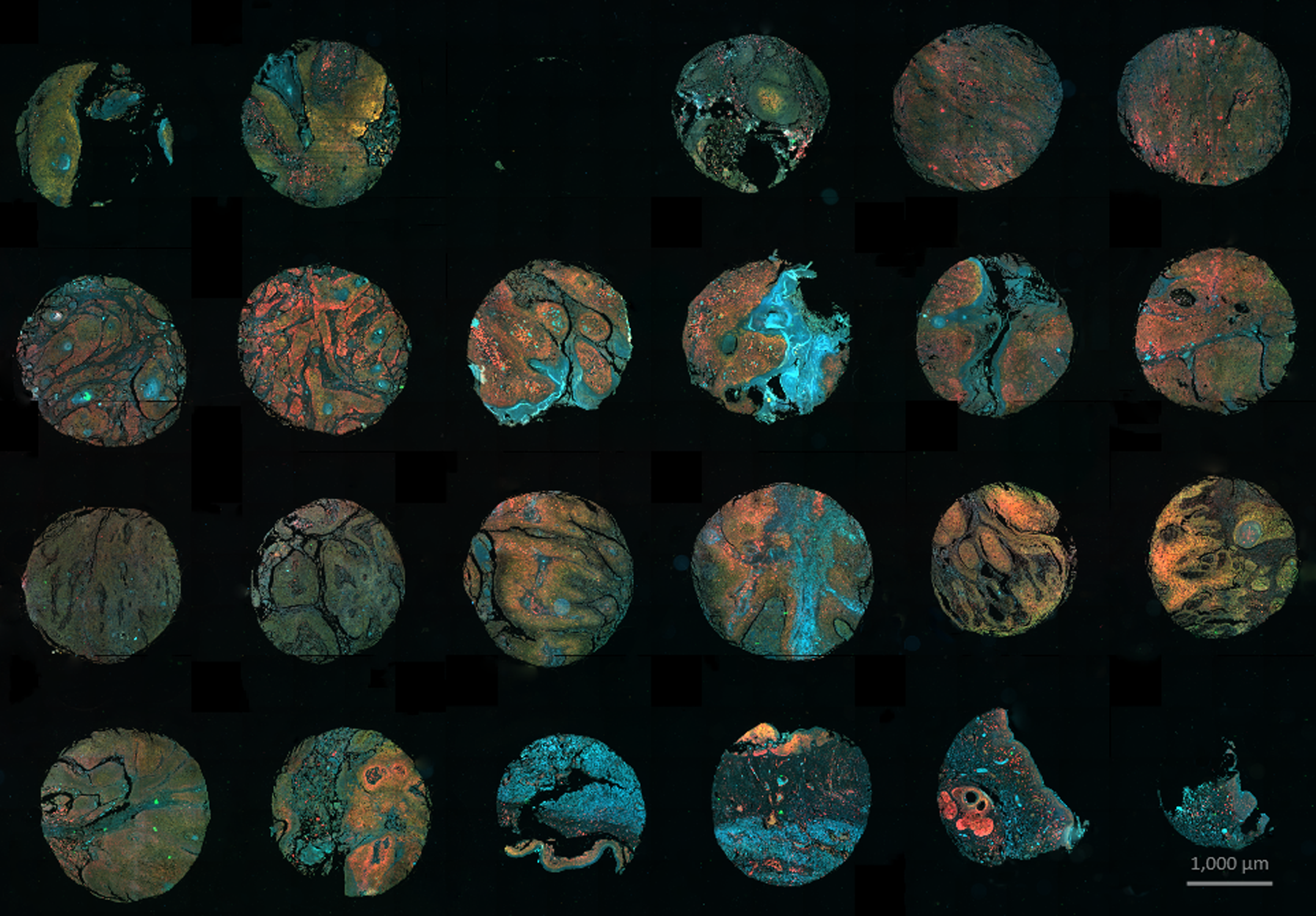

Supplement: S1 Fig — Tissue arrays were stained with four different antibodies and imaged using Zeiss AxioScan Z1 slide scanner. Composite, overlay, of four fluorophores (FITC, Alexa Fluor-405, Cy3 and Cy5 for MMP7, Wnt4, CD1 and c-MYC proteins, respectively) are shown. (TIF) [file pone.0124395.s001.tif]

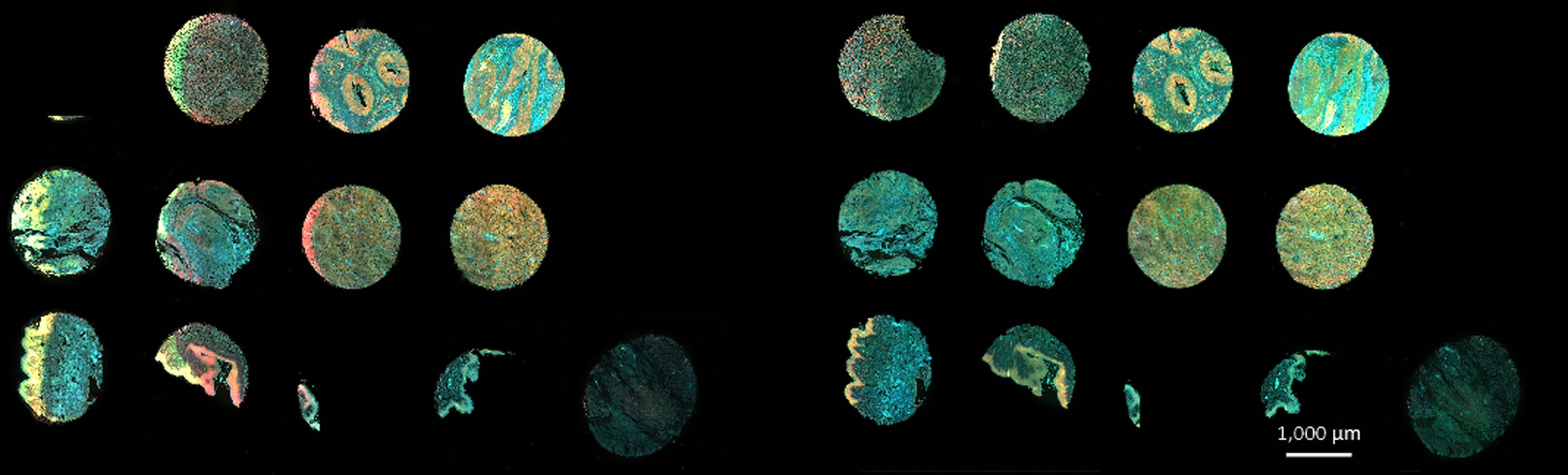

Supplement: S2 Fig — Tissue arrays were stained with four different antibodies and imaged using Zeiss AxioScan Z1 slide scanner. Composite, overlay, of four fluorophores (FITC, Alexa Fluor-405, Cy3 and Cy5 for MMP7, Wnt4, CD1 and c-MYC proteins, respectively) are shown. (TIF) [file pone.0124395.s002.tif]
